# Blood and Serum Copper and Zinc Levels and 10-Year Survival of Patients After Kidney Cancer Diagnosis

**DOI:** 10.3390/nu17060944

**Published:** 2025-03-08

**Authors:** Elżbieta Złowocka-Perłowska, Piotr Baszuk, Wojciech Marciniak, Róża Derkacz, Aleksandra Tołoczko-Grabarek, Katarzyna Gołębiewska, Marcin Słojewski, Adam Gołąb, Artur Lemiński, Michał Soczawa, Rodney J. Scott, Jan Lubiński

**Affiliations:** 1 Department of Genetics and Pathology, International Hereditary Cancer Center, Pomeranian Medical University, 70-204 Szczecin, Poland; piotr.baszuk@pum.edu.pl (P.B.); otjg@interia.pl (A.T.-G.); k.tutlewska@wp.pl (K.G.); jan.lubinski@pum.edu.pl (J.L.); 2 Read-Gene, Grzepnica, ul. Alabastrowa 8, 72-003 Dobra, Poland; wojciech.marciniak@read-gene.com (W.M.); roza.derkacz@read-gene.com (R.D.); 3 Department of Urology and Oncological Urology Clinic, Pomeranian Medical University, 70-204 Szczecin, Poland; marcin@slojewski.com (M.S.); adam.golab@pum.edu.pl (A.G.); michal.soczawa@gmail.com (M.S.); 4 Department of Biochemical Research, Pomeranian Medical University, 70-204 Szczecin, Poland; artur.leminski@gmail.com; 5 School of Biomedical Sciences and Pharmacy, Centre for Information-Based Medicine, Hunter Medical Research Institute, University of Newcastle, Newcastle, NSW 2305, Australia; rodney.scott@newcastle.edu.au; 6 Division of Molecular Medicine, Pathology North, NSW Pathology, Newcastle, NSW 2305, Australia

**Keywords:** kidney cancer, survival, copper, zinc

## Abstract

**Background/Objectives:** Copper (Cu) and zinc (Zn) are essential trace elements, and an imbalance in their levels may influence the progression of cancer. The role of Cu and Zn levels in blood and serum, as well as 10-year survival rates in kidney cancer patients, remains unclear. Our objective was to determine the association between these micronutrients and mortality of kidney cancer patients. In this prospective study, we examined 284 consecutive, unselected kidney cancer patients and assessed their 10-year survival in relation to Cu and Zn levels. **Methods**: Micronutrient levels were measured using an inductively coupled plasma mass spectrometer. Each patient was categorized into one of four groups based on the distribution of Cu and Zn levels, ranked in increasing order. The multivariable models included factors such as age at diagnosis, gender, smoking history, type of surgery, and histopathological results. **Results**: We observed a significantly higher risk of all-cause mortality in patients with the highest blood or serum copper levels compared to those with the lower levels (blood: HR = 4.89; *p* < 0.001; serum: HR = 3.75; *p* < 0.001). With regard to zinc, we found a trend where lower blood or serum zinc levels (I quartile) were associated with higher mortality. Additionally, we identified a significant correlation between the Zn/Cu ratio and mortality. **Conclusions:** Patients in the lowest Zn/Cu ratio quartile had elevated hazard ratios compared to those in the higher quartile with HRs of 3.05 (*p* < 0.002) in blood and 5.72 (*p* < 0.001) in serum. To our knowledge, this study is the first to investigate the relationship between blood and serum levels of copper and zinc and kidney cancer survival.

## 1. Introduction

Kidney cancers account for approximately 2–3% of the global cancer burden [1]. More than 50% of kidney cancers worldwide are diagnosed incidentally in asymptomatic individuals during investigations for other conditions [2]. The 5-year cancer-specific survival rates for patients diagnosed with stage I and stage IV renal cell carcinoma are 83% and 6%, respectively [3].

In a previous report, we showed that selenium (Se) levels, combined selenium and zinc levels (SeQI-ZnQI vs. SeQIV-ZnQIV), and the zinc-to-selenium ratio (Zn/Se) presented a promising target for clinical trials aimed at improving survival in kidney cancer patients. Patients with the lowest selenium and zinc levels had significantly higher all-cause mortality compared to those with high selenium and zinc levels [4]. Research by Lubinski et al. has shown a link between high copper (Cu) levels and increased overall mortality in patients with breast, prostate, lung, and laryngeal cancer [5]. Szwiec et al. found that in breast cancer patients, a high copper/zinc ratio was associated with reduced overall 10-year survival and breast cancer-specific survival [6]. Similarly, Bengtsson et al. revealed that a higher copper/zinc ratio negatively impacted survival in breast cancer patients [7]. They observed a trend of lower breast cancer survival associated with higher copper and lower zinc levels. Therefore, it seems reasonable to examine copper and zinc levels and the zinc-to-copper ratio (Zn/Cu) as well as 10-year survival in patients following a kidney cancer diagnosis.

Research suggests that maintaining optimal levels of these trace elements may have significant anti-cancer effects [8,9]. Copper and zinc are essential trace elements that play important roles in numerous physiological processes. Copper plays a critical role in free radical detoxification, cellular respiration, neurotransmitter synthesis, and various biological processes including the regulation of sex hormones, and excess copper is known to contribute to tumorigenesis, angiogenesis, and metastasis; by silencing oncogenes, it can restore the expression of suppressor genes [10,11,12,13,14,15,16]. The role of copper in the body is crucial for energy production in mitochondria, protection against free radicals, and the synthesis of neurotransmitters. Copper is a cofactor of superoxide dismutase (Cu, Zn-SOD), which protects cells from oxidative stress [17]. Elevated copper levels have been observed in both tumor tissue and serum [18,19]. Copper drives coordinated metabolic reprogramming, enhancing bioenergetics, biosynthesis, and redox homeostasis, which collectively promote tumor growth [19].

In turn, zinc is essential in many metabolic processes, participates in the synthesis of proteins and hormones and affects the functioning of the immune system. It is a cofactor for many enzymes and affects the synthesis of DNA and RNA [20]. Zinc plays a vital role in maintaining normal growth, cell proliferation, DNA repair, apoptosis, protein synthesis, and overall cellular homeostasis [21]. Several studies have shown that zinc deficiency in patients with breast, gastric, colorectal, lung, and prostate cancer and leukemia correlates with disease progression and lower survival rates [22,23].

The relationship between zinc and copper in the human body is complex. An elevated serum Cu/Zn ratio has been shown to be potentially useful in cancer diagnosis, prognosis, tumor staging, and predicting overall patient survival [24].

To date, there are no reports correlating copper and zinc levels in blood and serum with the survival of kidney cancer patients.

The aim of the project is to evaluate the association between copper and zinc levels in blood and serum and kidney cancer mortality.

## 2. Materials and Methods

### 2.1. Study Group

This study includes 284 unselected kidney cancer cases diagnosed at the Urology and Oncological Urology Clinic, University Hospital in Szczecin between 2014 and 2017. All eligible patients provided written informed consent for blood/serum samples to be collected specifically for research purposes. The blood and serum samples were taken at the same time from each patient between 8 a.m. and 11 a.m. at the time of diagnosis but before the start of treatment. They were stored in an existing research biobank at −80 °C until analysis. In analysis of elements, levels the above storage conditions do not change measurement reliability. All patients were asked to fast for six hours before the blood/serum samples were collected. The cases were not selected based on age, sex, smoking status, type of surgery, histological features, or, if applicable, cause of death. Detailed characteristics of the study group are presented in Table 1. Information on vital status and date of death was obtained from the Polish Ministry of Internal Affairs and Administration in November 2023. This study was approved by the Ethics Committee of Pomeranian Medical University in Szczecin, under the reference number KB-006/07/22.

### 2.2. Sample Collection and Storage

Two venous blood samples (10 cm^3^ each) were collected from each patient. One sample was transferred into an EDTA (ethylene–diaminetetraacetic acid) tube and stored at −80 °C until analysis. The other sample was transferred to a Beckton Dickinson Vacutainer tube (Beckton Dickinson, Franklin Lakes, NJ, USA) with a clot activator and incubated at room temperature for at least 30 min. It was then centrifuged at 1300× *g* for 12 min, and the serum was stored at −80 °C. On the day of analysis, both the blood sample and serum were thawed, mixed by vortexing, and centrifuged at 5000× *g* for 5 min.

### 2.3. Measurement Methodology

Micronutrient levels of copper (Cu) and zinc (Zn) in blood and serum were measured using an inductively coupled plasma mass spectrometer (ICP-MS) (ELAN DRC-e, Perkin Elmer, Woodbridge, ON, Canada). Before each analysis, the spectrometer was calibrated with external calibration standards. Calibration standards for Cu and Zn (1; 2; 3; 4; 5; 10; 50; 75; 100; 120; 150; and 170 µg/L) were prepared fresh daily by diluting a 10 µg/mL Multi-Element Calibration Standard 3 (PerkinElmer Pure Plus, Shelton, CT, USA) with a blank reagent. Oxygen was used as the reaction gas and the correlation coefficients for calibration curves were consistently greater than 0.999. The analysis protocol involved a 40-fold dilution of the serum with a blank reagent made from high-purity water (>18 MΩ), TMAH (Alfa Aesar, Tewksbury, MA, USA), Triton X-100 (PerkinElmer, Springfield, IL, USA), n-butanol (Merck, Darmstadt, Germany), and EDTA (Sigma-Aldrich, St. Louis, MO, USA). Technical details are available upon request.

### 2.4. Quality Control

The accuracy and precision of all measurements were tested using certified reference material (CRM), Clincheck Plasmonorm Blood Trace Elements Level 1 (Recipe, Munich, Germany). Technical details, plasma operating settings, and mass spectrometer acquisition parameters can be provided upon request.

### 2.5. Statistical Analysis

To estimate the relationship between blood and serum copper and zinc levels and kidney cancer survival, univariable and multivariable Cox regression models were used. Blood and serum levels of copper and zinc were categorized into four subgroups (quartiles) based on their concentrations, arranged in ascending order. The quartile with the lowest observed number of deaths was chosen as the reference range for each element analyzed. An observation period of ≥10 years was treated as a 10-year follow-up in all calculations. In the multivariable models, the following variables were considered: age at diagnosis (≤60/>60), sex (female/male), smoking status (no/current–former smoker), type of surgery (nephrectomy/tumorectomy), Fuhrman grade (G I-IV), and histopathological results (clear cell/papillary–chromophobe). A *p*-value ≤ 0.05 was considered statistically significant, while Kaplan–Meier curves were used to illustrate the association between the analyzed quartiles of elements and survival in kidney cancer patients. All calculations were performed using the R environment (R Foundation for Statistical Computing, Vienna, Austria 2023; R version: 4.3.2).

## 3. Results

**Figure 1:**
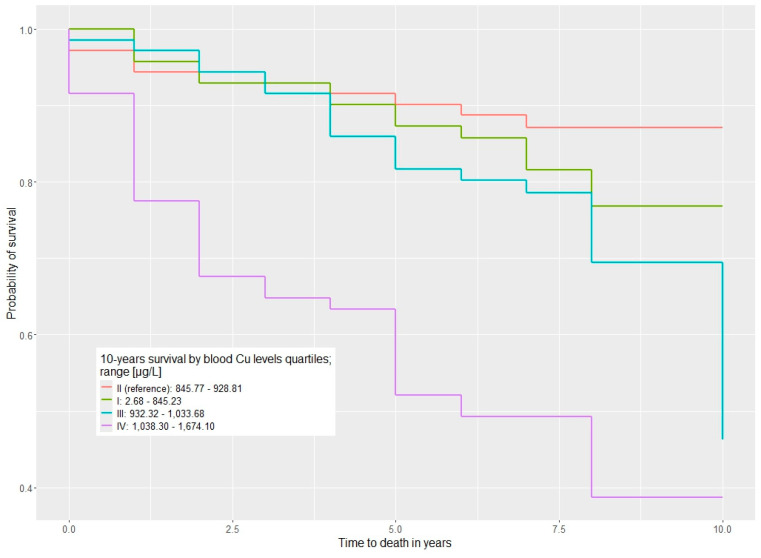
Ten-year overall survival by Cu blood level (µg/L) categorized into quartiles (QI–QIV).

Characteristics of Cu levels in blood/serum and ten-year survival for univariable and multivariable Cox proportional hazard regression models are detailed in Table 2 and Table 3 and the survival distributions are visualized with Kaplan–Meier curves in Figure 1. The correlations between the Zn/Cu ratio in blood/serum and ten-year survival outcomes are presented in Table 4 and Table 5.

### 3.1. Copper

#### 3.1.1. Copper in Blood

Copper blood levels appear to be associated with the survival rate of kidney cancer patients, as shown in Table 2. The subgroup of patients with the highest copper levels (IV quartile) had significantly higher all-cause mortality compared to those with lower copper levels (II quartile), with a hazard ratio (HR) of 4.89 (*p* < 0.001). We observed that the association with all-cause mortality was stronger for women than for men. Women with the highest blood copper levels had an HR of 6.48 (*p* = 0.019) (Supplementary Material Table S1), while men had an HR of 2.74 (*p* = 0.017) comparing the highest and lowest copper levels (Supplementary Material Table S2).

Further analysis of death due to kidney cancer progression revealed a strong association. Patients in quartile IV had a significantly higher all-cause mortality compared to those in quartile II, with an HR of 12.8 (*p* < 0.001) (Supplementary Material Table S3). This correlation was particularly strong among women, with an HR of 8.67 (*p* = 0.04) (Supplementary Material Table S4), whereas the association in men was HR = 3.79, *p* = 0.03 (Supplementary Material Table S5).

**Table 2:** Correlation between Cu levels in blood and all-cause mortality of kidney cancer patients.

	Vital Status			Univariable COX Regression			Multivariable COX Regression		
Variables	Overall *n* = 284 ^1^	Alive *n* = 204 ^1^	Deceased *n* = 80 ^1^	HR ^2^	95% CI ^2^	*p*-Value	HR ^2^	95% CI ^2^	*p*-Value
Cu									
II (reference) 845.77–928.81	71 (25%)	62 (30%)	9 (11%)	—	—		—	—	
I: 2.68–845.23	71 (25%)	58 (28%)	13 (16%)	1.55	0.66–3.63	0.3	1.25	0.53–2.98	0.6
III: 932.32–1033.68	71 (25%)	52 (25%)	19 (24%)	2.19	0.99–4.85	0.052	2.24	1.01–4.98	0.049
IV: 1038.30–1674.10	71 (25%)	32 (16%)	39 (49%)	6.16	2.98–12.7	<0.001	4.89	2.27–10.5	<0.001

^1^ *n* (%); ^2^ HR = hazard ratio; CI = confidence interval.

#### 3.1.2. Copper in Serum

Similar results were observed when analyzing serum copper levels. The hazard ratios increased significantly with higher copper levels, with an HR of 3.75 (*p* < 0.001) (Table 3). Among women, the HR for those in the highest quartile of copper levels compared to the lower was 7.47 (*p* = 0.01), while for men, it was 5.38 (*p* = 0.001) (Supplementary Material Tables S6 and S7).

In the entire group of patients with high serum copper levels, the HR for death due to disease progression was 6.96 (*p* = 0.002) (Supplementary Material Table S8). In women, this HR was higher than in men (HR = 18.4, *p* = 0.008 vs. HR = 6.04, *p* = 0.01) (Supplementary Material Table S9 and S10).

**Table 3:** Correlation between Cu levels in serum and all-cause mortality of kidney cancer patients.

	Vital Status			Univariable COX Regression			Multivariable COX Regression		
Variables	Overall *n* = 284 ^1^	Alive *n* = 204 ^1^	Deceased *n* = 80 ^1^	HR ^2^	95% CI ^2^	*p*-Value	HR ^2^	95% CI ^2^	*p*-Value
Cu									
II (reference): 1077.44–1217.10	71 (25%)	60 (29%)	11 (14%)	—	—		—	—	
I: 518.09–1074.31	71 (25%)	57 (28%)	14 (18%)	1.26	0.57–2.77	0.6	0.96	0.43–2.14	>0.9
III: 1221.73–1411.25	71 (25%)	54 (26%)	17 (21%)	1.55	0.72–3.30	0.3	1.58	0.72–3.45	0.3
IV: 1413.75–2422.49	71 (25%)	33 (16%)	38 (48%)	4.67	2.39–9.15	<0.001	3.75	1.80–7.79	<0.001

^1^ *n* (%); ^2^ HR = hazard ratio; CI = confidence interval.

### 3.2. Zinc

#### 3.2.1. Zinc in Blood

The association between blood zinc levels and all-cause mortality in kidney cancer patients showed that the subgroup with lowest zinc concentrations had higher hazard ratios compared to patients with higher zinc levels. However, statistical significance was not achieved in the multivariable Cox regression analysis (Supplementary Material Table S11).

Further analysis taking into account gender revealed that in the male subgroup, individuals in quartile III had significantly lower all-cause mortality compared to those in quartile I, with an HR of 2.95 (*p* = 0.008) (Supplementary Material Table S12). In women, this difference was not statistically significant. The HR in blood was 0.82 (*p* = 0.7) (Supplementary Material Table S13). Further analysis in the subgroup of men revealed that in the third quartile, there were fewer deaths caused by kidney cancer progression compared to the first quartile, with an HR of 4.79 (*p* = 0.01) (Supplementary Material Table S14).

#### 3.2.2. Zinc in Serum

The analysis of the association between serum zinc levels and all-cause mortality in kidney cancer patients suggested that patients with lower zinc concentrations had higher hazard ratios compared to those with higher zinc levels. However, this association did not reach statistical significance in the multivariable Cox regression analysis (Supplementary Material, Table S15).

Gender-stratified analysis revealed that in male patients, those in the third quartile of serum zinc levels had lower all-cause mortality compared to those in the first quartile, with an HR of 0.70 (*p* = 0.4) (Supplementary Material, Table S16). Among female patients, no statistically significant differences were observed. In the serum zinc analysis, the lowest all-cause mortality was noted in the second quartile (HR 1.90, *p* = 0.4) (Supplementary Material, Table S17). Further investigation within the male subgroup revealed a trend toward reduced kidney cancer-related mortality in the third quartile compared to the first quartile, with an HR of 3.02 (*p* = 0.06) (Supplementary Material, Table S18).

### 3.3. CuQIV-ZnQIV vs. CuQI-ZnQI

#### 3.3.1. CuQIV-ZnQIV vs. CuQI-ZnQI in Blood

In this study, we did not identify any synergistic effects between copper and zinc levels. Patients in the highest quartiles for both copper and zinc (CuQIV-ZnQIV) had a hazard ratio for all-cause mortality of 3.59 compared to those in the lowest quartiles (CuQI-ZnQI), though this result did not reach statistical significance (*p* = 0.06). Among men, the HR was notably higher at 6.15, but this too did not achieve statistical significance (*p* = 0.09) (Supplementary Material, Tables S19 and S20). We observed that, for all patients, the HR for death due to kidney cancer progression was 6.32 (*p* = 0.08), compared to an HR of 1.56 (*p* = 0.7) for death due to other causes (Supplementary Material, Tables S21 and S22).

#### 3.3.2. CuQIV-ZnQIV vs. CuQI-ZnQI in Serum

When serum copper and zinc levels were combined (CuQIV-ZnQIV vs. CuQI-ZnQI), the hazard ratio for all-cause mortality was 2.03, which was not statistically significant (*p* = 0.2). Among men, the HR was similarly 1.59 (*p* = 0.5) (Supplementary Material, Tables S23 and S24). No significant differences were observed between deaths due to kidney cancer progression and death from other causes (HR = 2.61, *p* = 0.3 vs. HR = 3.73, *p* = 0.2) (Supplementary Material, Tables S25 and S26).

### 3.4. Zn/Cu Ratio

#### 3.4.1. Zn/Cu Ratio in Blood

Our data revealed that patients with the lowest blood Zn/Cu ratio had an HR of 3.05 (*p* < 0.002) compared to those with the highest Zn/Cu ratio (Table 4). This correlation was also significant among men, with an HR of 3.16 (*p* = 0.007) (Supplementary Material, Table S27). Furthermore, our findings indicated that mortality due to kidney cancer progression was higher in patients with the lowest Zn/Cu ratios in blood (HR = 6.56, *p* < 0.001) (Supplementary Material, Table S28). Specifically, men in the first quartile had a significantly higher risk of death due to kidney cancer progression compared to those in the fourth quartile, HR of 3.95 (*p* = 0.04), Supplementary Material, Table S29. Among women, a similar trend was observed, but the correlation did not reach statistical significance, HR = 6.20, (*p* = 0.1), Supplementary Material, Table S30. Of note, among men, the HR for death due to other causes was notably higher, HR = 9.41 (*p* = 0.039), Supplementary Material, Table S31.

**Table 4:** Survival of kidney cancer patients according to blood Zn/Cu ratio.

	Vital Status			Univariable COX Regression			Multivariable COX Regression		
Variables	Overall *n* = 284 ^1^	Alive *n* = 204 ^1^	Deceased *n* = 80 ^1^	HR ^2^	95% CI ^2^	*p*-Value	HR ^2^	95% CI ^2^	*p*-Value
Zn/Cu									
III (reference): 6.88–7.78	71 (25%)	58 (28%)	13 (16%)	—	—		—	—	
I: 0.25–5.78	71 (25%)	39 (19%)	32 (40%)	3.20	1.68–6.12	<0.001	3.05	1.52–6.11	0.002
II: 5.81–6.87	71 (25%)	51 (25%)	20 (25%)	1.51	0.75–3.03	0.3	1.51	0.74–3.06	0.3
IV: 7.78–10.97	71 (25%)	56 (27%)	15 (19%)	1.21	0.57–2.54	0.6	0.93	0.44–1.98	0.9

^1^ *n* (%); ^2^ HR = hazard ratio; CI = confidence interval.

#### 3.4.2. Zn/Cu Ratio in Serum

In serum, we observed a much stronger correlation between the lowest Zn/Cu ratio and mortality compared to blood, with those in the highest Zn/Cu ratio group (HR = 5.72, *p* ≤ 0.001) (Table 5). This correlation was even stronger among women (HR = 8.28, *p* = 0.008) compared to men (HR = 6.50, *p* = 0.001) (Supplementary Material, Tables S32 and S33). Our data also revealed that patients with a low Zn/Cu ratios had a significantly higher risk of death due to kidney cancer progression compared to those with a high Zn/Cu ratio, HR = 11.5, (*p* = 0.001), Supplementary Material, Table S34.

**Table 5:** Survival of kidney cancer patients according to serum Zn/Cu ratio.

	Vital Status			Univariable COX Regression			Multivariable COX Regression		
Variables	Overall *n* = 284 ^1^	Alive *n* = 204 ^1^	Deceased *n* = 80 ^1^	HR ^2^	95% CI ^2^	*p*-Value	HR ^2^	95% CI ^2^	*p*-Value
Zn/Cu									
IV (reference) 0.84–1.48	71 (25%)	63 (31%)	8 (10%)	—	—		—	—	
I: 0.00–0.57	71 (25%)	35 (17%)	36 (45%)	6.15	2.86–13.3	<0.001	5.72	2.50–13.1	<0.001
II: 0.57–0.70	71 (25%)	52 (25%)	19 (24%)	2.61	1.14–5.97	0.023	2.69	1.16–6.23	0.021
III: 0.70–0.84	71 (25%)	54 (26%)	17 (21%)	2.18	0.94–5.05	0.069	2.28	0.98–5.32	0.057

^1^ *n* (%); ^2^ HR = hazard ratio; CI = confidence interval.

## 4. Discussion

In the current study, we examined 10-year survival of kidney cancer patients in relation to blood/serum copper and zinc levels. We found that patients with the highest copper levels and lowest zinc levels had significantly higher all-cause mortality. This study revealed a significant association between a low Zn/Cu ratio and increased mortality.

We observed that patients with low blood/serum copper levels had significantly better survival rates compared to those with higher levels.

This study investigated the relationship between blood and serum zinc levels and all-cause mortality in kidney cancer patients. The findings revealed that patients with lower zinc concentrations had higher hazard ratios (HRs) compared to those with higher zinc levels.

Examining the Zn/Cu ratio, we observed that a higher zinc/copper ratio was associated with significantly better survival rates.

What is of particular value is that analysis of the Zn/Cu ratio in both whole blood and serum allows for the identification of subgroups of patients (~20%, ~40%) with relatively low Cu levels but high risk of progression because of low Zn levels and thus Zn/Cu ratio.

The relationship between overall survival or cancer progression and blood/serum copper and zinc levels in kidney cancer patients is not well documented in the literature. However, a similar observation was made by Mimata et al. in 1986, who evaluated 100 patients with gastric cancer and found a correlation between high serum copper levels and survival, though no correlation was found with histological type or liver metastases [25]. Later reports by Wang et al. confirmed that high serum copper levels were progressively elevated in advanced gastric cancer [26]. Several other studies have shown similar correlations. Fang et al. followed 989 patients with hepatocellular carcinoma for four years and found that low serum copper was associated with better survival [27]. Recent work by our group, involving 1475 patients with breast, prostate, lung, and laryngeal cancer, showed a significant correlation between low copper levels and better overall survival across all cancer types [5]. In a prospective cohort study of 1998 breast cancer patients, Bengtsson et al. observed a trend between higher serum copper levels and decreased breast cancer survival [7]. Similarly, Zabłocka-Słowińska et al., in a study of 167 lung cancer patients, showed that higher serum copper levels were associated with increased all-cause mortality [28]. Leone et al. investigated 4035 men, who volunteered for cardiovascular screening, and found that high copper levels were associated with a 40% increase in cancer mortality and the combination of high copper and low zinc levels increased cancer mortality risk [29].

Wu et al. found that, in a group of 3244 women and 3000 men who were cancer-free, those with lower serum copper levels had a reduced risk of dying from cancer [30]. Additionally, patients with mycosis fungoides who had higher serum copper levels were found to have a higher risk of disease progression and poorer survival than those with normal copper levels [31]. In contrast, Xianghui et al. found that higher serum copper concentrations in adult Americans were not associated with increased cancer-related mortality [32].

The evidence presented herein and by others generally reveals elevated copper levels in various malignancies impacting the survival of cancer patients. Defective copper metabolism can lead to disturbances in copper homeostasis, contributing to disease progression [33]. Bischoff et al. showed that late-stage clear cell renal cell carcinoma accumulates copper and directs it to mitochondrial cytochrome c oxidase [19]. By activating metabolic and proliferative enzymes and especially lysine oxidase, copper plays a major role in cancer cell metastasis [34,35].

In terms of blood/serum zinc levels and 10-year survival following a kidney cancer diagnosis, we observed a trend towards higher zinc concentrations being linked to lower all-cause mortality compared to lower zinc levels (as noted in our previous publication) [4]. This observation is consistent with other studies on breast, stomach, colon, leukemia, lung, laryngeal, and prostate cancers [5,7,22,23,24,36]. Pietrzak et al. investigated 338 prostate cancer patients and observed that low zinc levels in serum, correlated with higher mortality in men [37]. In contrast, Fang et al. found no significant associations between serum zinc levels and survival or disease recurrence in hepatocellular carcinoma [27].

In the literature, there are few studies showing the relationship between copper and zinc, as well as their impact on overall survival and cancer progression. Published evidence highlights the zinc-to-copper ratio (Zn/Cu) as a potential prognostic biomarker, especially in women with breast cancer. A study involving 583 women with breast cancer showed that a higher Zn/Cu ratio was linked to improved survival rates [6]. Matuszczak et al. observed a correlation in women with a BRCA1 mutation. Those with a Zn/Cu ratio greater than 6.38 experienced a significantly lower cancer risk compared to those with a ratio below this threshold [38]. Bengtsson et al. found in women with breast cancer that subgroup with the highest copper/zinc levels had significantly higher all-cause mortality [7]. Fang et al. observed that in patients with hepatocellular carcinoma, the copper/zinc ratio was positively associated with liver cancer-specific survival and overall survival [27]. Kudva et al. noted an increase in the copper/zinc ratio among individuals with head and neck cancer [39]. Ito et al. observed no overall association between the copper/zinc ratio and cancer mortality in 507 individuals from rural Japan [40]. Mimata et al. examined patients with gastric cancer and identified an association between an elevated copper/zinc ratio and survival outcomes [25]. Wang et al. corroborated these findings, demonstrating that the copper/zinc ratio increased progressively in advanced stages of gastric cancer [26]. There are a few studies suggesting an adverse association—Tamai et al. studied 175 patients with hepatocellular carcinoma and found that the copper/zinc ratio was a strong predictor of survival. Patients with a Cu/Zn ratio ≥ 0.999 exhibited significantly higher survival rates compared to those with a Cu/Zn ratio < 0.999 [41]. Hastuti et al. examined 40 hematological malignancy patients and showed a decrease in copper isotopes and an increase in zinc isotopes associated with significantly poorer survival (HR 3.9) [42].

It is well accepted that an imbalance between copper and zinc leads to increased cellular oxidative stress and DNA damage and inflammation, which can lead to the development of tumors [43]. Zinc and copper can affect each other’s absorption and metabolism. The concentration of Zn and Cu in serum is regulated by the transfer of these elements from the intestinal lumen to the portal circulation [44]. A high Cu/Zn ratio is a risk factor for disability and mortality in elderly individuals, as it may reflect the body’s response to the accumulation of genotoxic stress [45,46]. An imbalance between copper and zinc levels can lead to tumorigenesis due to the overproduction of reactive oxygen species (ROS) [47].

Despite important findings, our study has several limitations: First, it was conducted in a single medical center, so future multi-center validation studies will be important. Second, the sample size was relatively small, with only 284 kidney cancer patients. Nevertheless, this is the first study to examine the effect of blood/serum copper levels and the zinc/copper ratio on survival in kidney cancer patients. The blood and serum samples were collected from the same patients at diagnosis but prior to treatment. These data provide a strong foundation for collaboration with other scientists to confirm our findings. If our results are confirmed by additional studies, then it can be considered to use levels of copper and zinc not only as prognostic markers but also as targets to be optimized by diet and/or supplementation in order to improve survival of kidney cancer patients.

## 5. Conclusions

In summary, the current findings showed that in kidney cancer patients, the lowest blood/serum copper levels were associated with a reduced risk of death. A low Zn/Cu ratio correlated with increased mortality. These findings suggest that the balance of copper and zinc may significantly impact the risk of mortality. Our analyses indicate that if copper levels are not too high, zinc can neutralize the effects of copper, leading to a significant decrease in mortality. Therefore, optimizing the blood/serum Zn/Cu ratio by increasing blood/serum zinc levels and reducing blood/serum copper levels might be justified and potentially serve as a procedure to improve survival.

## 6. Patents

Based on the results presented in this paper, a patent application has been submitted to the Patent Office of the Republic of Poland (P. 451230).

## Figures and Tables

**Table 1:** Characteristics of the patients in this study.

Variables	Overall *n* = 284	Alive *n* = 204	Dead *n* = 80
Age of diagnosis (mean)			
≤60 (50.12)	121 (43%)	98 (48%)	23 (29%)
>61 (67.66)	163 (57%)	106 (52%)	57 (71%)
Sex			
Female	118 (42%)	91 (45%)	27 (34%)
Male	166 (58%)	113 (55%)	53 (66%)
Smoking status			
No	95 (33%)	77 (38%)	18 (23%)
Current/former smoker	189 (67%)	127 (62%)	62 (78%)
Kind of operation			
Nephrectomy	126 (44%)	84 (41%)	42 (53%)
Tumorectomy	158 (56%)	120 (59%)	38 (48%)
Histological features			
* GI	75 (26%)	64 (31%)	11 (14%)
GII	125 (44%)	96 (47%)	29 (36%)
GIII	63 (22%)	39 (19%)	24 (30%)
GIV	21 (7.4%)	5 (2.5%)	16 (20%)
Clear cell carcinoma	245 (86%)	169 (83%)	76 (95%)
Papillary/chromophobe	39 (14%)	35 (17%)	4 (5.0%)
Death due to cancer			
No	-	-	19 (29%)
Yes	-	-	46 (71%)
Unknown	-	-	15

* GI–GIV—Fuhrman grade.

## Data Availability

Our data contain potentially sensitive information; therefore, we have not included them with our manuscript. Those who would like to request access to our data may contact Melissa Sidhu at the Research Ethics Board of the Women’s College Hospital by calling +1-(416)-351-3732 (ext. 2723) or emailing ac.latipsohcw@uhdis.assilem. The Pomeranian University of Medicine Ethics Committee will grant access to all researchers who meet the criteria for access to confidential data.

## Supplementary Materials

The following supporting information can be downloaded at: https://www.mdpi.com/article/10.3390/nu17060944/s1, Table S1. Survival of kidney cancer females according to blood Cu levels; Table S2. Survival of kidney cancer in males according to blood Cu levels; Table S3. Survival of kidney cancer patients according to blood Cu levels among kidney cancer-specific death; Table S4. Survival of kidney cancer women according to blood Cu levels among kidney cancer-specific death; Table S5. Survival of kidney cancer men according to blood Cu levels among kidney cancer-specific death; Table S6. Survival of kidney cancer women according to serum Cu levels; Table S7. Survival of kidney cancer men according to serum Cu levels; Table S8. Survival of kidney cancer patients according to serum Cu levels among kidney cancer-specific death; Table S9. Survival of kidney cancer women according to serum Cu levels among kidney cancer-specific death; Table S10. Survival of kidney cancer men according to serum Cu levels among kidney cancer-specific death; Table S11. Correlation between Zn levels in blood and all-cause mortality of kidney cancer patients; Table S12. Survival of kidney cancer men according to blood Zn levels; Table S13. Survival of kidney cancer women according to blood Zn levels; Table S14. Survival of kidney cancer men according to blood Zn levels among kidney cancer-specific death; Table S15. Correlation between Zn levels in serum and all-cause mortality of kidney cancer patients; Table S16. Survival of kidney cancer men according to serum Zn levels; Table S17. Survival of kidney cancer women according to serum Zn levels; Table S18. Survival of kidney cancer men according to serum Zn levels among kidney cancer-specific death; Table S19. Survival of kidney cancer patients according to blood combined effect of Cu and Zn levels by quartiles (CuQIV-ZnQIV vs. CuQI-ZnQI); Table S20. Survival of kidney cancer men according to blood combined effect of Cu and Zn levels by quartiles (CuQIV-ZnQIV vs. CuQI-ZnQI); Table S21. Survival of kidney cancer patients according to blood combined effect of Cu and Zn levels by quartiles (CuQIV-ZnQIV vs. CuQI-ZnQI) among kidney cancer-specific death; Table S22. Survival of kidney cancer patients according to blood combined effect of Cu and Zn levels by quartiles (CuQIV-ZnQIV vs. CuQI-ZnQI) among non-cancer-specific death; Table S23. Survival of kidney cancer patients according to serum combined effect of Cu and Zn levels by quartiles (CuQIV-ZnQIV vs. CuQI-ZnQI); Table S24. Survival of kidney cancer men according to serum combined effect of Cu and Zn levels by quartiles (CuQIV-ZnQIV vs. CuQI-ZnQI); Table S25. Survival of kidney cancer patients according to serum combined effect of Cu and Zn levels by quartiles (CuQIV-ZnQIV vs. CuQI-ZnQI) among kidney cancer-specific death; Table S26. Survival of kidney cancer patients according to serum combined effect of Cu and Zn levels by quartiles (CuQIV-ZnQIV vs. CuQI-ZnQI) among non-cancer-specific death; Table S27. Survival of kidney cancer men according to blood Zn/Cu ratio; Table S28. Survival of kidney cancer patients according to blood Zn/Cu ratio among kidney cancer-specific death; Table S29. Survival of male kidney cancer patients according to blood Zn/Cu ratio among kidney cancer-specific death; Table S30. Survival of kidney cancer women according to blood Zn/Cu ratio among kidney cancer-specific death; Table S31. Survival of male kidney cancer patients according to blood Zn/Cu ratio among non-cancer-specific death; Table S32. Survival of female kidney cancer women patients according to serum Zn/Cu ratio; Table S33. Survival of male kidney cancer patients according to serum Zn/Cu ratio; Table S34. Survival of kidney cancer patients according to serum Zn/Cu ratio among kidney cancer-specific death.
