# Supplementary material for: Blood and Serum Copper and Zinc Levels and 10-Year Survival of Patients After Kidney Cancer Diagnosis
Source: Nutrients. 2025 Mar 8;17(6):944. doi: 10.3390/nu17060944 (PMC11944747; doi:10.3390/nu17060944)
Supplement: Supplementary file 1 [file nutrients-17-00944-s001.zip › nutrients-3515743-supplementary.pdf]

Table S1. Survival of kidney cancer females according to blood Cu levels.

	Vital status			Univariable COX Regression			Multivariable COX Regression		
**Variables**	**Overall N=118[1]**	**Alive N=91[1]**	**Deceased N=27[1]**	**HR[2]**	**95% CI[2]**	***p*-value**	**HR[2]**	**95% CI[2]**	***p*-value**
Cu									
I (reference) 795.90 - 910.62 (859.93)	30 (25%)	28 (31%)	2 (7.4%)	-	-		-	-	
II 911.18 - 974.01 (943.12)	29 (25%)	29 (32%)	0 (0%)	-	-		-	-	
III 980.00 - 1125.06 (1031.88)	29 (25%)	20 (22%)	9 (33%)	5.15	1.11 23.8	0.036	5.82	1.24 27.3	0.026
IV 1141.58 - 1647.93 (1336.64)	30 (25%)	14 (15%)	16 (59%)	10.7	2.47 46.7	0.002	6.48	1.36 30.9	0.019

[1]n (%),[2]HR = Hazard Ratio, CI = Confidence Interval

Table S2. Survival of kidney cancer in males according to blood Cu levels.

	Vital status			Univariable COX Regression			Multivariable COX Regression		
**Variables**	**Overall N=166[1]**	**Alive N=113[1]**	**Deceased N=53[1]**	**HR[2]**	**95% CI[2]**	***p*-value**	**HR[2]**	**95% CI[2]**	***p*-value**
Cu									
I (reference) 2.68 - 818.42 (745.91)	42 (25%)	34 (30%)	8 (15%)	—	—		—	—	
II 820.36 - 889.90 (851.47)	41 (25%)	33 (29%)	8 (15%)	0.94	0.35 2.51	>0.9	1.03	0.38 2.77	>0.9
III 894.05 - 988.28 (937.52)	41 (25%)	28 (25%)	13 (25%)	1.56	0.65 3.79	0.3	1.61	0.65 3.96	0.3
IV 992.56 - 1674.10 (1146.62)	42 (25%)	18 (16%)	24 (45%)	3.79	1.70 8.45	0.001	2.74	1.19 6.29	0.017

[1]n (%),[2]HR = Hazard Ratio, CI = Confidence Interval

Table S3. Survival of kidney cancer patients according to blood Cu levels among kidney cancer-specific death.

	Vital status			Univariable COX Regression			Multivariable COX Regression		
**Variables**	**Overall N=250**[1]	**Alive N=204**[1]	**Deceased N=46**[1]	**HR**[2]	**95% CI**[2]	***p*-value**	**HR**[2]	**95% CI**[2]	***p*-value**
Cu									
II(reference) 843.76 - 925.53	62 (25%)	60 (29%)	2 (4.3%)	—	—		—	—	
I: 2.68 - 843.08	63 (25%)	57 (28%)	6 (13%)	3.14	0.63 15.6	0.2	2.76	0.55 13.8	0.2
III: 925.63 - 1033.28	62 (25%)	55 (27%)	7 (15%)	3.62	0.75 17.4	0.11	3.57	0.73 17.4	0.12
IV: 1038.30 - 1674.10	63 (25%)	32 (16%)	31 (67%)	21.5	5.13 89.9	<0.001	12.8	2.91 56.2	<0.001

[1]n (%),[2]HR = Hazard Ratio, CI = Confidence Interval

Table S4. Survival of kidney cancer women according to blood Cu levels among kidney cancer-specific death.

	Vital status			Univariable COX Regression			Multivariable COX Regression		
**Variables**	**Overall N=110[1]**	**Alive N=91[1]**	**Deceased N=19[1]**	**HR[2]**	**95% CI[2]**	***p*-value**	**HR[2]**	**95% CI[2]**	***p*-value**
Cu									
I (reference) 795.90 - 908.73 (858.23)	28 (25%)	27 (30%)	1 (5.3%)	—	—		—	—	
II 910.62 - 967.59 (938.66)	27 (25%)	27 (30%)	0 (0%)	-	-		-	-	
III 971.62 - 1141.58 (1034.83)	27 (25%)	23 (25%)	4 (21%)	4.34	0.48 38.8	0.2	4.04	0.44 37.3	0.2
IV 1153.33 - 1647.93 (1340.98)	28 (25%)	14 (15%)	14 (74%)	18.3	2.41 139	0.005	8.67	1.02 74.0	0.048

[1]n (%),[2]HR = Hazard Ratio, CI = Confidence Interval

Table S5. Survival of kidney cancer men according to blood Cu levels among kidney cancer-specific death.

	Frequency of deaths			Univariable COX Regression			Multivariable COX Regression		
**Variables**	**Overall N=140[1]**	**Alive N=113[1]**	**Deceased N=27[1]**	**HR[2]**	**95% CI[2]**	***p*-value**	**HR[2]**	**95% CI[2]**	***p*-value**
Cu									
II (reference) 816.61 - 874.19	35 (25%)	32 (28%)	3 (11%)	—	—		—	—	
I: 2.68 - 815.70	35 (25%)	31 (27%)	4 (15%)	1.40	0.31 6.25	0.7	1.58	0.34 7.25	0.6
III: 874.98 - 983.36	35 (25%)	32 (28%)	3 (11%)	1.00	0.20 4.94	>0.9	1.09	0.22 5.45	>0.9
IV: 988.28 - 1674.10	35 (25%)	18 (16%)	17 (63%)	7.61	2.22 26.0	0.001	3.79	1.08 13.3	0.038

[1]n (%),[2]HR = Hazard Ratio, CI = Confidence Interval

Table S6. Survival of kidney cancer women according to serum Cu levels.

	Vital status			Univariable COX Regression			Multivariable COX Regression		
**Variables**	**Overall N=118[1]**	**Alive N=91[1]**	**Deceased N=27[1]**	**HR[2]**	**95% CI[2]**	***p*-value**	**HR[2]**	**95% CI[2]**	***p*-value**
Cu									
II (reference) 1180.53 - 1282.71	29 (25%)	27 (30%)	2 (7.4%)	—	—		—	—	
I: 858.08 - 1178.83	30 (25%)	27 (30%)	3 (11%)	1.47	0.24 8.77	0.7	1.10	0.18 6.80	>0.9
III: 1284.59 - 1523.68	29 (25%)	25 (27%)	4 (15%)	2.11	0.39 11.5	0.4	1.80	0.32 10.1	0.5
IV: 1536.75 - 2422.49	30 (25%)	12 (13%)	18 (67%)	12.7	2.94 54.8	<0.001	7.47	1.59 35.1	0.011

[1]n (%),[2]HR = Hazard Ratio, CI = Confidence Interval

Table S7. Survival of kidney cancer men according to serum Cu levels.

	Vital status			Univariable COX Regression			Multivariable COX Regression		
Variables	Overall N=166[1]	Alive N=113[1]	Deceased N=53[1]	HR[2]	95% CI[2]	*p*-value	HR[2]	95% CI[2]	*p*-value
Cu									
II(reference) 1034.31 - 1127.90	41 (25%)	36 (32%)	5 (9.4%)	—	—		—	—	
I: 518.09 - 1032.35	42 (25%)	33 (29%)	9 (17%)	1.83	0.61 5.45	0.3	1.83	0.60 5.59	0.3
III: 1130.44 - 1319.76	41 (25%)	26 (23%)	15 (28%)	3.13	1.14 8.61	0.027	3.15	1.13 8.83	0.029
IV: 1325.88 - 2331.57	42 (25%)	18 (16%)	24 (45%)	6.70	2.55 17.6	<0.001	5.38	1.97 14.7	0.001

[1]n (%),[2]HR = Hazard Ratio, CI = Confidence Interval

Table S8. Survival of kidney cancer patients according to serum Cu levels among kidney cancer-specific death.

	Vital status			Univariable COX Regression			Multivariable COX Regression		
**Variables**	**Overall N=250**[1]	**Alive N=204**[1]	**Deceased N=46**[1]	**HR**[2]	**95% CI**[2]	***p*-value**	**HR**[2]	**95% CI**[2]	***p*-value**
Cu									
II(reference) 1078.70 - 1217.10	62 (25%)	59 (29%)	3 (6.5%)	—	—		—	—	
I: 518.09 - 1077.44	63 (25%)	58 (28%)	5 (11%)	1.64	0.39 6.85	0.5	1.27	0.30 5.41	0.7
III: 1221.73 - 1405.04	62 (25%)	54 (26%)	8 (17%)	2.74	0.73 10.3	0.14	2.46	0.64 9.46	0.2
IV: 1413.75 - 2422.49	63 (25%)	33 (16%)	30 (65%)	13.2	4.03 43.3	<0.001	6.96	2.00 24.2	0.002

[1]n (%),[2]HR = Hazard Ratio, CI = Confidence Interval

Table S9. Survival of kidney cancer women according to serum Cu levels among kidney cancer-specific death.

	Vital status			Univariable COX Regression			Multivariable COX Regression		
**Variables**	**Overall N=110[1]**	**Alive N=91[1]**	**Deceased N=19[1]**	**HR[2]**	**95% CI[2]**	***p*-value**	**HR[2]**	**95% CI[2]**	***p*-value**
Cu									
I (reference) 904.14 - 1178.83 (1080.37)	28 (25%)	27 (30%)	1 (5.3%)	—	—		—	—	
II 1180.53 - 1282.71 (1238.99)	27 (25%)	27 (30%)	0 (0%)	-	-		-	-	
III 1284.59 - 1536.75 (1407.41)	27 (25%)	25 (27%)	2 (11%)	2.10	0.19 23.2	0.5	2.19	0.20 24.3	0.5
IV 1582.69 - 2422.49 (1866.57)	28 (25%)	12 (13%)	16 (84%)	23.3	3.08 176	0.002	18.4	2.14 159	0.008

[1]n (%),[2]HR = Hazard Ratio, CI = Confidence Interval

Table S10. Survival of kidney cancer men according to serum Cu levels among kidney cancer-specific death.

	Vital status			Univariable COX Regression			Multivariable COX Regression		
**Variables**	**Overall N=140[1]**	**Alive N=113[1]**	**Deceased N=27[1]**	**HR[2]**	**95% CI[2]**	***p*-value**	**HR[2]**	**95% CI[2]**	***p*-value**
Cu									
II (reference) 1032.35 - 1114.38	35 (25%)	33 (29%)	2 (7.4%)	—	—		—	—	
I: 518.09 - 1026.69	35 (25%)	32 (28%)	3 (11%)	1.52	0.25 9.12	0.6	1.99	0.32 12.3	0.5
III: 1115.32 - 1287.62	35 (25%)	29 (26%)	6 (22%)	3.10	0.62 15.3	0.2	2.80	0.55 14.3	0.2
IV: 1290.17 - 2331.57	35 (25%)	19 (17%)	16 (59%)	11.0	2.51 47.7	0.001	6.04	1.34 27.2	0.019

[1]n (%),[2]HR = Hazard Ratio, CI = Confidence Interval

Table S11. Correlation between Zn levels in blood and all-cause mortality of kidney cancer patients.

	Vital status			Univariable COX Regression			Multivariable COX Regression		
**Variables**	**Overall, N=284[1]**	**Alive N=204[1]**	**Deceased N=80[1]**	**HR[2]**	**95% CI[2]**	***p*-value**	**HR[2]**	**95% CI[2]**	***p*-value**
Zn									
III(reference) 6375.10 - 6943.56	71 (25%)	59 (29%)	12 (15%)	—	—		—	—	
I: 0.68 - 5708.67	71 (25%)	46 (23%)	25 (31%)	2.41	1.21 4.81	0.012	2.47	1.21 5.00	0.012
II: 5724.69 - 6374.45	71 (25%)	51 (25%)	20 (25%)	1.79	0.87 3.65	0.11	1.81	0.88 3.75	0.11
IV: 6946.63 - 9206.50	71 (25%)	48 (24%)	23 (29%)	2.16	1.08 4.35	0.030	1.91	0.94 3.87	0.072

[1]n (%),[2]HR = Hazard Ratio, CI = Confidence Interval

Table S12. Survival of kidney cancer men according to blood Zn levels.

	Vital status			Univariable COX Regression			Multivariable COX Regression		
**Variables**	**Overall N=166**[1]	**Alive N=113**[1]	**Deceased N=53**[1]	**HR**[2]	**95% CI**[2]	***p*-value**	**HR**[2]	**95% CI**[2]	***p*-value**
Zn									
III (reference) 6547.57 - 7043.41	41 (25%)	32 (28%)	9 (17%)	—	—		—	—	
I: 0.68 - 5891.19	42 (25%)	23 (20%)	19 (36%)	2.47	1.11 5.47	0.026	2.95	1.32 6.60	0.008
II: 5897.92 - 6509.50	41 (25%)	31 (27%)	10 (19%)	1.18	0.48 2.90	0.7	1.46	0.58 3.68	0.4
IV: 7089.13 - 9206.50	42 (25%)	27 (24%)	15 (28%)	1.84	0.81 4.21	0.15	1.93	0.84 4.43	0.12

[1]n (%),[2]HR = Hazard Ratio, CI = Confidence Interval

Table S13. Survival of kidney cancer women according to blood Zn levels.

	Vital status			Univariable COX Regression			Multivariable COX Regression		
**Variables**	**Overall N=118**[1]	**Alive N=91**[1]	**Deceased N=27**[1]	**HR**[2]	**95% CI**[2]	***p*-value**	**HR**[2]	**95% CI**[2]	***p*-value**
Zn									
II (reference): 5481.35 - 6068.35	29 (25%)	23 (25%)	6 (22%)	—	—		—	—	
I: 3321.34 - 5477.97	30 (25%)	22 (24%)	8 (30%)	1.32	0.46 3.81	0.6	0.82	0.26 2.58	0.7
III: 6074.69 - 6701.90	29 (25%)	23 (25%)	6 (22%)	1.00	0.32 3.09	>0.9	0.72	0.22 2.41	0.6
IV: 6786.62 - 8895.02	30 (25%)	23 (25%)	7 (26%)	1.17	0.39 3.48	0.8	1.00	0.33 3.07	>0.9

[1]n (%),[2]HR = Hazard Ratio, CI = Confidence Interval

Table S14. Survival of kidney cancer men according to blood Zn levels among kidney cancer-specific death.

	Frequency of deaths			Univariable COX Regression			Multivariable COX Regression		
**Variables**	**Overall N=140[1]**	**Alive N=113[1]**	**Deceased N=27[1]**	**HR[2]**	**95% CI[2]**	***p*-value**	**HR[2]**	**95% CI[2]**	***p*-value**
Zn									
III (reference): 6547.57 - 7042.98	35 (25%)	31 (27%)	4 (15%)	—	—		—	—	
I: 0.68 - 5922.33	35 (25%)	24 (21%)	11 (41%)	3.26	1.04 10.3	0.043	4.79	1.41 16.2	0.012
II: 5933.00 - 6509.50	35 (25%)	30 (27%)	5 (19%)	1.34	0.36 4.99	0.7	2.29	0.54 9.62	0.3
IV: 7043.41 - 9030.73	35 (25%)	28 (25%)	7 (26%)	1.93	0.56 6.58	0.3	2.48	0.71 8.66	0.2

[1]n (%),[2]HR = Hazard Ratio, CI = Confidence Interval

Table S15. Correlation between Zn levels in serum and all-cause mortality of kidney cancer patients.

	Vital status			Univariable Cox Regression			Multivariable Cox Regression		
**Variables**	**Overall N=284[1]**	**Alive N=204[1]**	**Deceased N=80[1]**	**HR[2]**	**95% CI[2]**	***p*-value**	**HR[2]**	**95% CI[2]**	***p*-value**
Zn									
IV(reference) 951.99 - 1280.44	71 (25%)	57 (28%)	14 (18%)	—	—		—	—	
I: 0.36 - 755.99	71 (25%)	41 (20%)	30 (38%)	2.64	1.40 4.98	0.003	1.92	0.98 3.75	0.055
II: 756.27 - 847.72	71 (25%)	53 (26%)	18 (23%)	1.38	0.69 2.78	0.4	1.30	0.62 2.70	0.5
III: 848.02 - 948.98	71 (25%)	53 (26%)	18 (23%)	1.37	0.68 2.75	0.4	1.13	0.55 2.32	0.7

[1]n (%),[2]HR = Hazard Ratio, CI = Confidence Interval

Table S16. Survival of kidney cancer men according to serum Zn levels.

	Vital status			Univariable COX Regression			Multivariable COX Regression		
**Variables**	**Overall N=166[1]**	**Alive N=113[1]**	**Deceased N=53[1]**	**HR[2]**	**95% CI[2]**	***p*-value**	**HR[2]**	**95% CI[2]**	***p*-value**
Zn									
IV (reference): 986.55 - 1280.44	42 (25%)	33 (29%)	9 (17%)	—	—		—	—	
I: 470.82 - 763.44	42 (25%)	22 (19%)	20 (38%)	2.75	1.25 6.06	0.012	1.63	0.68 3.91	0.3
II: 763.88 - 859.15	41 (25%)	28 (25%)	13 (25%)	1.67	0.71 3.90	0.2	1.50	0.59 3.84	0.4
III: 860.45 - 976.15	41 (25%)	30 (27%)	11 (21%)	1.23	0.51 2.96	0.6	0.70	0.28 1.76	0.4

[1]n (%),[2]HR = Hazard Ratio, CI = Confidence Interval

Table S17. Survival of kidney cancer women according to serum Zn levels.

	Vital status			Univariable COX Regression			Multivariable COX Regression		
**Variables**	**Overall N=118[1]**	**Alive N=91[1]**	**Deceased N=27[1]**	**HR[2]**	**95% CI[2]**	***p*-value**	**HR[2]**	**95% CI[2]**	***p*-value**
Zn									
IV (reference): 914.10 - 1213.44	30 (25%)	26 (29%)	4 (15%)	—	—		—	—	
I: 0.36 - 747.92	30 (25%)	19 (21%)	11 (41%)	3.41	1.08 10.7	0.036	2.24	0.66 7.63	0.2
II: 750.72 - 829.79	29 (25%)	24 (26%)	5 (19%)	1.33	0.36 4.94	0.7	1.90	0.49 7.41	0.4
III: 832.12 - 911.42	29 (25%)	22 (24%)	7 (26%)	2.04	0.60 6.96	0.3	2.00	0.56 7.19	0.3

[1]n (%),[2]HR = Hazard Ratio, CI = Confidence Interval

Table S18. Survival of kidney cancer men according to serum Zn levels among kidney cancer-specific death.

	Vital status			Univariable COX Regression			Multivariable COX Regression		
**Variables**	**Overall N=140[1]**	**Alive N=113[1]**	**Deceased N=27[1]**	**HR[2]**	**95% CI[2]**	***p*-value**	**HR[2]**	**95% CI[2]**	***p*-value**
Zn									
III (reference): 861.98 - 991.03	35 (25%)	31 (27%)	4 (15%)	—	—		—	—	
I: 470.82 - 763.88	35 (25%)	23 (20%)	12 (44%)	3.30	1.06 10.3	0.039	3.02	0.94 9.72	0.064
II: 770.05 - 860.45	35 (25%)	28 (25%)	7 (26%)	1.78	0.52 6.10	0.4	2.70	0.73 10.0	0.14
IV: 991.38 - 1280.44	35 (25%)	31 (27%)	4 (15%)	0.97	0.24 3.86	>0.9	2.20	0.50 9.70	0.3

[1]n (%),[2]HR = Hazard Ratio, CI = Confidence Interval

Table S19. Survival of kidney cancer patients according to blood combined effect of Cu and Zn levels by quartiles (CuQIV-ZnQIV vs. CuQI-ZnQI).

	Vital status			Univariable COX Regression			Multivariable COX Regression		
**Variables**	**Overall N=284**[1]	**Alive N=204**[1]	**Deceased N=80**[1]	**HR**[2]	**95% CI**[2]	***p*-value**	**HR**[2]	**95% CI**[2]	***p*-value**
CuQIV-ZnQIV vs. CuQI-ZnQI									
CuQIZnQI (reference): 2.68 - 845.23	12 (4.2%)	9 (4.4%)	3 (3.8%)	—	—		—	—	
CuQIVZnQIV 1038.30 - 1456.64	15 (5.3%)	6 (2.9%)	9 (11%)	3.30	0.89 12.2	0.073	3.59	0.94 13.7	0.061
Other 623.14 - 1674.10	257 (90%)	189 (93%)	68 (85%)	1.07	0.34 3.41	>0.9	1.18	0.36 3.87	0.8

[1]n (%),[2]HR = Hazard Ratio, CI = Confidence Interval

Table S20. Survival of kidney cancer men according to blood combined effect of Cu and Zn levels by quartiles *(*CuQIV-ZnQIV vs. CuQI-ZnQI).

	Vital status			Univariable COX Regression			Multivariable COX Regression		
**Variables**	**Overall N=166[1]**	**Alive N=113[1]**	**Deceased N= 3[1]**	**HR[2]**	**95% CI[2]**	***p*-value**	**HR[2]**	**95% CI[2]**	***p*-value**
CuQIV-ZnQIV vs. CuQI-ZnQI									
CuQ1ZnQ1 (reference): 2.68 - 795.16	8 (4.8%)	7 (6.2%)	1 (1.9%)	—	—		—	—	
CuQ4ZnQ4 998.14 – 1456.64	11 (6.6%)	3 (2.7%)	8 (15%)	8.20	1.02 65.7	0.047	6.15	0.74 50.9	0.092
Other: 623.14 - 1674.10	147 (89%)	103 (91%)	44 (83%)	2.31	0.32 16.8	0.4	1.92	0.26 14.3	0.5

[1]n (%),[2]HR = Hazard Ratio, CI = Confidence Interval

Table S21. Survival of kidney cancer patients according to blood combined effect of Cu and Zn levels by quartiles (CuQIV-ZnQIV vs. CuQI-ZnQI) among kidney cancer-specific death.

	Vital status			Univariable COX Regression			Multivariable COX Regression		
**Variables**	**Overall N=250**[1]	**Alive N=204**[1]	**Deceased N=46**[1]	**HR**[2]	**95% CI**[2]	***p*-value**	**HR**[2]	**95% CI**[2]	***p*-value**
CuQIV-ZnQIV vs. CuQI-ZnQI									
CuQ1ZnQ1. (reference): 2.68 - 838.16	10 (4.0%)	9 (4.4%)	1 (2.2%)	—	—		—	—	
CuQ4ZnQ4. 1038.30 – 1456.64	13 (5.2%)	6 (2.9%)	7 (15%)	7.87	0.97 64.0	0.054	6.32	0.76 52.7	0.088
Other: 623.14 - 1674.10	227 (91%)	189 (93%)	38 (83%)	1.66	0.23 12.1	0.6	1.93	0.26 14.6	0.5

[1]n (%),[2]HR = Hazard Ratio, CI = Confidence Interval

Table S22. Survival of kidney cancer patients according to blood combined effect of Cu and Zn levels by quartiles (CuQIV-ZnQIV vs. CuQI-ZnQI) among non cancer-specific death.

	Vital status			Univariable COX Regression			Multivariable COX Regression		
**Variables**	**Overall N=223[1]**	**Alive N=204[1]**	**Deceased N=19[1]**	**HR[2]**	**95% CI[2]**	***p*-value**	**HR[2]**	**95% CI[2]**	***p*-value**
CuQIV-ZnQIV vs. CuQI-ZnQI									
CuQ1ZnQ1 (reference): 2.68 - 835.29	9 (4.0%)	8 (3.9%)	1 (5.3%)	—	—		—	—	
CuQ4ZnQ4. 999.32 - 1410.04	11 (4.9%)	9 (4.4%)	2 (11%)	1.40	0.13 15.5	0.8	1.56	0.13 19.1	0.7
Other: 623.14 - 1532.94	203 (91%)	187 (92%)	16 (84%)	0.55	0.07 4.15	0.6	0.54	0.07 4.40	0.6

[1]n (%),[2]HR = Hazard Ratio, CI = Confidence Interval

Table S23. Survival of kidney cancer patients according to serum combined effect of Cu and Zn levels by quartiles (CuQIV-ZnQIV vs. CuQI-ZnQI).

	Vital status			Univariable COX Regression			Multivariable COX Regression		
**Variables**	**Overall N=284**[1]	**Alive N=204**[1]	**Deceased N=80**[1]	**HR**[2]	**95% CI**[2]	***p*-value**	**HR**[2]	**95% CI**[2]	***p*-value**
CuQIV-ZnQIV vs. CuQI-ZnQI									
CuQIZnQI (reference) 518.09 - 1074.31	21 (7.4%)	14 (6.9%)	7 (8.8%)	—	—		—	—	
CuQIVZnQIV 1435.73 - 2029.66	11 (3.9%)	5 (2.5%)	6 (7.5%)	2.06	0.69 6.13	0.2	2.03	0.66 6.29	0.2
Other: 621.93 - 2422.49	252 (89%)	185 (91%)	67 (84%)	0.85	0.39 1.86	0.7	1.00	0.45 2.23	>0.9

[1]n (%),[2]HR = Hazard Ratio, CI = Confidence Interval

Table S24. Survival of kidney cancer men according to serum combined effect of Cu and Zn levels by quartiles (CuQIV-ZnQIV vs. CuQI-ZnQI).

	Vital status			Univariable COX Regression			Multivariable COX Regression		
**Variables**	**Overall N=166[1]**	**Alive N=113[1]**	**Deceased N=53[1]**	**HR[2]**	**95% CI[2]**	***p*-value**	**HR**	**95% CI[2]**	***p*-value**
CuQIV-ZnQIV vs. CuQI-ZnQI									
CuQ1ZnQ1 (reference): 518.09 - 1025.85	11 (6.6%)	6 (5.3%)	5 (9.4%)	—	—		—	—	
CuQ4ZnQ4. 1326.88 - 2029.66	12 (7.2%)	6 (5.3%)	6 (11%)	1.19	0.36 3.92	0.8	1.59	0.44 5.83	0.5
Other: 621.93 - 2331.57	143 (86%)	101 (89%)	42 (79%)	0.64	0.25 1.63	0.4	0.64	0.24 1.68	0.4

[1]n (%),[2]HR = Hazard Ratio, CI = Confidence Interval

Table S25. Survival of kidney cancer patients according to serum combined effect of Cu and Zn levels by quartiles (CuQIV-ZnQIV vs. CuQI-ZnQI) among kidney cancer-specific death.

	Vital status			Univariable COX Regression			Multivariable COX Regression		
**Variables**	**Overall N=250**[1]	**Alive N=204**[1]	**Deceased N=46**[1]	**HR**[2]	**95% CI**[2]	***p*-value**	**HR**[2]	**95% CI**[2]	***p*-value**
CuQIV-ZnQIV vs. CuQI-ZnQI									
CuQ1ZnQ1. (reference): 518.09 - 1074.31	17 (6.8%)	14 (6.9%)	3 (6.5%)	—	—		—	—	
CuQ4ZnQ4. 1443.74 - 2029.66	8 (3.2%)	5 (2.5%)	3 (6.5%)	2.67	0.54 13.2	0.2	2.61	0.48 14.4	0.3
Other: 621.93 - 2422.49	225 (90%)	185 (91%)	40 (87%)	1.05	0.32 3.38	>0.9	0.99	0.30 3.33	>0.9

[1]n (%),[2]HR = Hazard Ratio, CI = Confidence Interval

Table S26. Survival of kidney cancer patients according to serum combined effect of Cu and Zn levels by quartiles (CuQIV-ZnQIV vs. CuQI-ZnQI) among non cancer-specific death.

	Vital status			Univariable COX Regression			Multivariable COX Regression		
**Variables**	**Overall N=223[1]**	**Alive N=204[1]**	**Deceased N=19[1]**	**HR[2]**	**95% CI[2]**	***p*-value**	**HR[2]**	**95% CI[2]**	***p*-value**
CuQIV-ZnQIV vs. CuQI-ZnQI									
CuQ1ZnQ1 (reference): 518.09 - 1058.81	14 (6.3%)	12 (5.9%)	2 (11%)	—	—		—	—	
CuQ4ZnQ4. 1333.44 - 2029.66	9 (4.0%)	7 (3.4%)	2 (11%)	1.46	0.20 10.5	0.7	3.73	0.42 33.2	0.2
Other: 621.93 - 2422.49	200 (90%)	185 (91%)	15 (79%)	0.53	0.12 2.31	0.4	0.72	0.16 3.25	0.7

[1]n (%),[2]HR = Hazard Ratio, CI = Confidence Interval

Table S27. Survival of kidney cancer men according to blood Zn/Cu ratio.

	Vital status			Univariable COX Regression			Multivariable COX Regression		
**Variables**	**Overall N=166[1]**	**Alive N=113[1]**	**Deceased N=53[1]**	**HR[2]**	**95% CI[2]**	***p*-value**	**HR[2]**	**95% CI[2]**	***p*-value**
Zn/Cu									
IV (reference): 8.10 - 10.97	42 (25%)	34 (30%)	8 (15%)	—	—		—	—	
I: 0.25 - 6.26	42 (25%)	20 (18%)	22 (42%)	3.56	1.58 8.01	0.002	3.16	1.37 7.29	0.007
II: 6.27 - 7.44	41 (25%)	27 (24%)	14 (26%)	1.90	0.80 4.53	0.15	1.67	0.68 4.08	0.3
III: 7.45 - 8.09	41 (25%)	32 (28%)	9 (17%)	1.23	0.47 3.20	0.7	1.13	0.42 3.05	0.8

[1]n (%),[2]HR = Hazard Ratio, CI = Confidence Interval

Table S28. Survival of kidney cancer patients according to blood Zn/Cu ratio among kidney cancer-specific death.

	Vital status			Univariable COX Regression			Multivariable COX Regression		
**Variables**	**Overall N=250**[1]	**Alive N=204**[1]	**Deceased N=46**[1]	**HR**[2]	**95% CI**[2]	***p*-value**	**HR**[2]	**95% CI**[2]	***p*-value**
Zn/Cu									
III (reference): 6.87 - 7.75	62 (25%)	57 (28%)	5 (11%)	—	—		—	—	
I: 0.25 - 5.61	63 (25%)	36 (18%)	27 (59%)	6.86	2.64 17.9	<0.001	6.56	2.27 18.9	<0.001
II: 5.63 - 6.87	62 (25%)	54 (26%)	8 (17%)	1.61	0.53 4.93	0.4	1.73	0.56 5.40	0.3
IV: 7.78 - 10.97	63 (25%)	57 (28%)	6 (13%)	1.22	0.37 3.99	0.7	1.09	0.33 3.65	0.9

[1]n (%),[2]HR = Hazard Ratio, CI = Confidence Interval

Table S29. Survival of male kidney cancer patients according to blood Zn/Cu ratio among kidney cancer-specific death.

	Vital status			Univariable COX Regression			Multivariable COX Regression		
**Variables**	**Overall N=140**[1]	**Alive N=113**[1]	**Deceased N=27**[1]	**HR**[2]	**95% CI**[2]	***p*-value**	**HR**[2]	**95% CI**[2]	***p*-value**
Zn/Cu									
IV (reference): 8.21 - 10.97	35 (25%)	32 (28%)	3 (11%)	—	—		—	—	
I: 0.25 - 6.30	35 (25%)	21 (19%)	14 (52%)	6.09	1.75 21.2	0.005	3.95	1.06 14.6	0.040
II: 6.33 - 7.46	35 (25%)	29 (26%)	6 (22%)	2.09	0.52 8.35	0.3	1.19	0.28 5.06	0.8
III: 7.50 - 8.15	35 (25%)	31 (27%)	4 (15%)	1.44	0.32 6.45	0.6	0.77	0.16 3.59	0.7

[1]n (%),[2]HR = Hazard Ratio, CI = Confidence Interval

Table S30. Survival of kidney cancer women according to blood Zn/Cu ratio among kidney cancer-specific death.

	Vital status			Univariable COX Regression			Multivariable COX Regression		
**Variables**	**Overall N=110[1]**	**Alive N=91[1]**	**Deceased N=19[1]**	**HR[2]**	**95% CI[2]**	***p*-value**	**HR[2]**	**95% CI[2]**	***p*-value**
Zn/Cu									
IV (reference): 7.02 - 9.68	28 (25%)	27 (30%)	1 (5.3%)	—	—		—	—	
I: 2.98 - 5.12	28 (25%)	16 (18%)	12 (63%)	14.1	1.84 109	0.011	6.20	0.71 54.4	0.10
II: 5.14 - 6.04	27 (25%)	23 (25%)	4 (21%)	4.32	0.48 38.7	0.2	3.31	0.33 33.0	0.3
III: 6.08 -7.00	27 (25%)	25 (27%)	2 (11%)	1.93	0.17 21.3	0.6	1.98	0.17 22.4	0.6

[1]n (%),[2]HR = Hazard Ratio, CI = Confidence Interval

Table S31. Survival of male kidney cancer patients according to blood Zn/Cu ratio among non cancer-specific death.

	Vital status			Univariable COX Regression			Multivariable COX Regression		
**Variables**	**Overall N=126[1]**	**Alive N=113[1]**	**Deceased N=13[1]**	**HR[2]**	**95% CI[2]**	***p*-value**	**HR[2]**	**95% CI[2]**	***p*-value**
Zn/Cu									
IV (reference): 8.25 - 10.97	32 (25%)	31 (27%)	1 (7.7%)	—	—		—	—	
I: 0.25 - 6.60	32 (25%)	25 (22%)	7 (54%)	7.00	0.85 57.4	0.070	9.41	1.12 79.3	0.039
II:6.67 - 7.55	31 (25%)	29 (26%)	2 (15%)	1.83	0.16 20.3	0.6	2.30	0.20 27.0	0.5
III: 7.56 -8.21	31 (25%)	28 (25%)	3 (23%)	3.15	0.33 30.4	0.3	5.38	0.52 55.7	0.2

[1]n (%),[2]HR = Hazard Ratio, CI = Confidence Interval

Table S32. Survival of female kidney cancer women patients according to serum Zn/Cu ratio.

	Vital status			Univariable COX Regression			Multivariable COX Regression		
**Variables**	**Overall N=118[1]**	**Alive N=91[1]**	**Deceased N=27[1]**	**HR[2]**	**95% CI[2]**	***p*-value**	**HR[2]**	**95% CI[2]**	***p*-value**
Zn/Cu									
IV(reference): 0.74 - 1.07	30 (25%)	28 (31%)	2 (7.4%)	—	—		—	—	
I: 0.00 - 0.50	30 (25%)	14 (15%)	16 (59%)	11.0	2.52 47.7	0.001	8.28	1.74 39.5	0.008
II: 0.51 - 0.63	29 (25%)	23 (25%)	6 (22%)	3.34	0.67 16.6	0.14	3.48	0.67 18.0	0.14
III: 0.63 -0.74	29 (25%)	26 (29%)	3 (11%)	1.60	0.27 9.59	0.6	2.00	0.33 12.2	0.5

[1]n (%),[2]HR = Hazard Ratio, CI = Confidence Interval

Table S33. Survival of male kidney cancer patients according to serum Zn/Cu ratio.

	Vital status			Univariable COX Regression			Multivariabl COX Regression		
**Variables**	**Overall N=166[1]**	**Alive N=113[1]**	**Deceased N=53[1]**	**HR[2]**	**95% CI[2]**	***p*-value**	**HR[2]**	**95% CI[2]**	***p*-value**
Zn/Cu									
IV(reference): 0.89 - 1.48	42 (25%)	38 (34%)	4 (7.5%)	—	—		—	—	
I: 0.21 - 0.62	42 (25%)	19 (17%)	23 (43%)	8.84	3.04 25.7	<0.001	6.50	2.13 19.9	0.001
II: 0.62 - 0.76	41 (25%)	25 (22%)	16 (30%)	4.66	1.56 14.0	0.006	3.12	1.02 9.58	0.047
III: 0.76 -0.88	41 (25%)	31 (27%)	10 (19%)	2.69	0.84 8.58	0.095	2.69	0.83 8.67	0.10

[1]n (%),[2]HR = Hazard Ratio, CI = Confidence Interval

Table S34. Survival of kidney cancer patients according to serum Zn/Cu ratio among kidney cancer-specific death.

	Vital status			Univariable COX Regression			Multivariable COX Regression		
**Variables**	**Overall N=250[1]**	**Alive N=204[1]**	**Deceased N=46[1]**	**HR[2]**	**95% CI[2]**	***p*-value**	**HR[2]**	**95% CI[2]**	***p*-value**
Zn/Cu									
IV (reference): 0.84 - 1.48	63 (25%)	61 (30%)	2 (4.3%)	—	—		—	—	
I: 0.21 - 0.56	63 (25%)	33 (16%)	30 (65%)	20.2	4.81 84.4	<0.001	11.5	2.57 51.8	0.001
II: 0.56 -0.70	62 (25%)	54 (26%)	8 (17%)	4.37	0.93 20.6	0.062	4.04	0.84 19.4	0.081
III: 0.70-0.84	62 (25%)	56 (27%)	6 (13%)	3.13	0.63 15.5	0.2	2.63	0.52 13.2	0.2

[1]n (%),[2]HR = Hazard Ratio, CI = Confidence Interval
